# Children with Cerebral Palsy Across the Gross Motor Function Classification System Levels Requiring Orthopaedic Surgery: The Lived Experiences of Parents †

**DOI:** 10.3390/children12101411

**Published:** 2025-10-18

**Authors:** Maria Juricic, Stacey D. Miller, Emily K. Schaeffer, Kishore Mulpuri, Lesley Bainbridge

**Affiliations:** 1 Department of Orthopaedic Surgery, BC Children’s Hospital, Vancouver, BC V6H 3V4, Canada; smiller4@cw.bc.ca (S.D.M.);; 2 Department of Physical Therapy, University of British Columbia, Vancouver, BC V6T 1Z3, Canada; 3 BC Children’s Hospital Research Institute, Vancouver, BC V5Z 4H4, Canada; 4 Department of Orthopaedics, University of British Columbia, Vancouver, BC V5Z 1M9, Canada

**Keywords:** disability, lived experiences, interprofessional, cerebral palsy, surgery, parenting

## Abstract

Background/Objectives: Orthopaedic surgery is often recommended for children with cerebral palsy (CP) across all Gross Motor Function Classification System (GMFCS) levels. Despite this, little is known about the experience of parents during their child’s surgery and recovery. Methods: This topic was explored using a mixed-methods research design. Using an interpretive description methodology, in-depth interviews with parents of children with CP who had undergone orthopaedic surgery were completed by a physical therapist within an interdisciplinary clinical context in an acute care orthopaedic surgery clinic. Transcripts were reviewed by inductive thematic analysis. Resulting themes were used to inform the development of a self-administered survey, which was distributed to a separate cluster sample of parents. Results: From interviews with six parents, four themes were identified: (1) preparing and being prepared, (2) feeling known and recognized, (3) knowing and advocating for your child, and (4) feeling stressed and coping. The results of surveys completed by 25 parents were analyzed using descriptive statistics. When asked whether their child’s surgery was a stressful experience, 80% (20/25) agreed. However, 60% (15/25) indicated that the surgery was not a negative experience. Forty-four percent (11/25) felt their child’s recovery was longer than expected. Survey responses to questions related to the qualitative themes were similar across GMFCS levels and surgical procedures. Conclusions: The findings identify the importance of recognizing the needs of parents and suggest opportunities for collaboration between the healthcare team and families in caring for children across the spectrum of functional mobility and orthopaedic procedures.

## 1. Introduction

Cerebral palsy (CP) is the most common chronic disorder and cause of disability among children [1], with a prevalence of 2.1 per 1000 births [2,3]. While the neurological impairments associated with CP are static by definition, the musculoskeletal complications are often progressive [4]. Children with CP may experience gait abnormalities, deformities, contractures, and pain; consequently, orthopaedic surgery may be recommended [5]. Surgery can involve bony procedures, for example, osteotomies for treatment of hip displacement or spinal fixation for scoliosis [6,7,8], and soft tissue procedures, such as tendon lengthening for contractures [9,10,11]. Surgery is directed at improving function and quality of life [1,5]. The evolving needs of the child with CP over the course of their growth and development often leads to ongoing orthopaedic surgical management.

Children with CP experience a wide range of surgical needs and functional mobility [12]. The Gross Motor Function Classification System (GMFCS) describes function based on the child’s ability for independent mobility [12]. It is a 5-level classification system routinely used by clinicians to describe, assess, and anticipate the needs of children, as relates to their functional mobility. Classification comprises a spectrum, which includes children at level I, who are independent in ambulation, with mild deficits in balance and coordination. Children at level III may use handheld assistive devices. Those at level V rely on wheeled mobility devices exclusively and have impairment in all motor functions. The mobility of those at levels II and IV falls between higher and lower levels accordingly. The 5-level classifications are also described in detail across five age bands, allowing for classification for children from ages 2–18. The GMFCS provides a clinical picture of a child’s mobility, facilitating communication and documentation. It is also useful in predicting the functional prognosis of children with CP [13] and in identifying those at greatest risk of orthopaedic complications [14,15]. While surgical recommendations are not determined by a child’s classification alone, common issues can be found among patients who share the same GMFCS level. This allows clinicians to anticipate what orthopaedic management children may require based on their GMFCS level. It is an essential clinical tool that can provide insight into the resource and care needs of children across the spectrum of CP and their families, as well as its use is standard practice for those caring for children with CP.

In their application of the World Health Organization International Classification of Function, Health, and Disability (ICF) model to children with CP, Rosenbaum and Steward [16] highlighted the need to consider the impact of social constructs or “environmental factors” and “personal factors”, which provide context to the experience of disability [17]. These factors include a child’s access to medical and rehabilitation interventions, demographics and interests, and the direct influence of the family context [16]. Parenting a child with CP has been associated with higher levels of stress and financial burden, as well as impacts on social, physical, and psychological health [18,19]. Family-centred care has long been identified as the preferred service delivery approach for children with chronic health conditions [20,21]. It promotes the well-being of both children and parents [22,23] through its pillars, including, shared decision making, partnership, information sharing, as well as flexibility, respect, support, and empowerment in service delivery [23]. Despite the frequency of orthopaedic procedures for children with CP, little is known about the experience of orthopaedic surgery for these children and their families.

Improving our understanding of the parental experience and how it is associated with their child’s functional mobility when using the GMFCS may assist healthcare practitioners in better anticipating and meeting the needs of all families in this position. The objective of this study was to explore the following question: what is the lived experience of parents of children with CP across the GMFCS levels who require orthopaedic surgery?

## 2. Materials and Methods

### 2.1. Design

**Figure 1:**
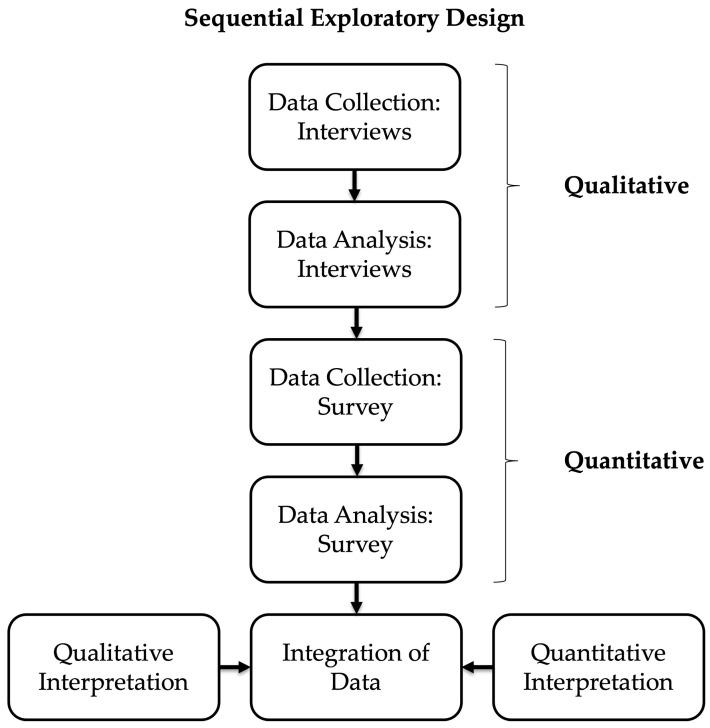
Outline of sequential exploratory study design, including collection and analysis of qualitative, then quantitative data, followed by integration of qualitative and quantitative data.

A sequential exploratory mixed-methods research (MMR) design was selected (Figure 1). Interpretive and constructivist theoretical frameworks recognize the presence of multiple subjective realities, whereby an individual’s interpretation of a shared or common experience is determined by the personal meaning attributed to the particular experience and may define and be defined by the interactions within an individual’s life [24]. Interpretive description is a qualitative methodology that seeks to uncover the elements, connections, and patterns within experience, the meaning of which may be understood and applied according to the perspective of a researcher’s clinical discipline [25]. Informed by these theoretical and methodological perspectives, interviews were performed by a physical therapist within an interdisciplinary clinical context in an acute care orthopaedic surgery clinic. Qualitative data from these interviews were collected and analyzed, the results of which were used to inform the development of a quantitative survey [26].

### 2.2. Participants: Interviews

Parents of children with CP under the age of 18 were eligible if their child had undergone orthopaedic surgery within the previous two years. Parents of children with underlying progressive neurological diagnoses or those with limited spoken English language abilities were not included. The University of British Columbia/Children’s and Women’s Health Centre of British Columbia Research Ethics Board approved the study (H15-00956, H15-03251). All patients provided written or electronic informed consent prior to participation in the study.

### 2.3. Recruitment: Interviews

Purposive sampling was used to recruit participants for in-depth interviews from the caseloads of orthopaedic surgeons at an urban tertiary care centre. An informal sampling frame was used to identify parents with varying surgical experience [27], stratified according to their child’s GMFCS level. This identified twenty potential parent participants, who were mailed a letter of invitation. Written informed consent was obtained by a research assistant from those parents who contacted the researcher expressing interest in participating in the study.

### 2.4. Data Collection: Interviews

Interviews were completed between June and October 2015. Interview questions were developed following a review of the related literature by the interviewing author, a physiotherapist with extensive experience with the study population and setting. This was done in consultation with the co-investigators, including an orthopaedic surgeon specializing in the care of children with neuromotor conditions. Questions were then piloted with a parent prior to the interviews. Examples of questions included the following: “From who do you usually get medical information to help meet your child’s needs?” “How did you prepare for surgery?” “As a parent, how did you cope through your child’s surgery process?” “Looking back, how did the actual experience compare to what you anticipated?” (See Supplementary Material, S1: Parent Inverview Script.) Parents were asked to confirm their child’s GMFCS level, if required using the the GMFCS Family Report Questionnaire [28,29,30]. Interviews were audio recorded and transcribed verbatim.

Interview transcripts were forwarded to each corresponding parent. A brief follow-up telephone interview was later completed by the interviewing author with each participant, for member checking, to ensure comments were correctly understood and recorded and to review the themes identified.

### 2.5. Qualitative Data Analysis

Qualitative data were analyzed by inductive thematic analysis [31,32]. This involved a step-by-step process of repeated reading of interview transcripts and detailed coding of data items. Coding involved identifying meaningful text segments, by interview question, as they related to the research question through the lens of the study’s theoretical frameworks and methodological approach. This was followed by compiling of codes across interviews, by question, to review for common codes, leading to the identification of initial areas of interest. (These areas of interest were subsequently used to inform the development of the quantitative survey.) Codes were further evaluated, grouped, and organized into subthemes to produce a thematic map to assist analysis and further interpretation in order to finalize the main themes [32]. A second researcher reviewed a sample of coded transcripts and thematic map development.

### 2.6. Recruitment: Survey

Survey participants were recruited by cluster sampling of parents accompanying children to scheduled visits in an outpatient orthopaedic department between March and May 2016. Inclusion and exclusion criteria were consistent with the qualitative study strand, with the exception that parents of children having undergone surgery in the past 5 years were considered, to facilitate recruitment. Thirty-one eligible participants were approached by a research assistant and invited to participate. Consenting parents used a tablet computer to complete the anonymous, self-administered online survey. Seven parents who were unable to complete the survey at the time of recruitment were provided information on accessing the online survey remotely.

### 2.7. Data Collection: Survey

Data collected included child and parent demographics, the GMFCS Family Report Questionnaire [28,29,30] (by age band), and questions related to the parent’s experience of the child’s surgery with respect to the initial areas of interest identified in the qualitative analysis. These areas of interest included the following: preparation, communication/accessing information, parent expertise, trust in relationships with healthcare provider, hope, and parent stress. Questions were developed as they related to decision making, hospitalization, and recovery, as assessed by a 7-point rating scale [33]. Survey results were collected using REDCap electronic data capture tools [34], hosted at the research institute associated with the tertiary care centre.

### 2.8. Quantitative Data Analysis

To provide additional insight into the results of the interviews [26,35], survey results were analyzed across all GMFCS levels and within two separate groups of predominately ambulatory children (GMFCS levels I, II, and III), and non-ambulatory children (GMFCS levels IV and V). Categorical data was presented in frequencies [35]. Given the small size of the sample and subgroups, only descriptive statistics were used. Survey data were compared with interview data and were integrated with qualitative findings in the final study analysis [35].

### 2.9. Integration of Qualitative and Quantitative Data

Following the qualitative and quantitative data collection and analysis, integration of data from each research strand occurred at the point of interpretation and writing of the manuscript [36]. This was achieved by merging qualitative and quantitative results through a process of drawing connections between themes or subthemes from the qualitative analysis and the results from individual or groups of survey questions and weaving them into the qualitative narrative as they related to the themes [37]. While prioritizing qualitative data, quantitative data were used to expand and refine the interpretation of the themes in describing the experiences of participating parents [36,37]. The sequence of data collection, analysis, and integration is represented in Figure 1.

## 3. Results

**Figure 2:**
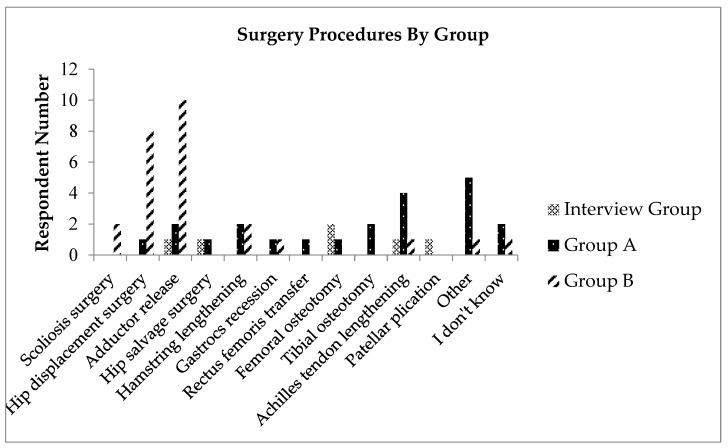
Surgery procedures received by children of parents in the interview group, survey Group A, and survey Group B.

Of the 20 parents invited to participate in the study, in-depth interviews were completed with 6 consenting parents (5 children). All children had a diagnosis of CP, and all levels of the GMFCS were represented. Data from 25 completed surveys were analyzed and presented in two groups. Group A included survey results from parents of children classified at GMFCS levels I, II, and III, and Group B from parents of children classified at GMFCS levels IV and V. Demographic information of qualitative and quantitative study strands are reported in Table 1, and the surgical procedures undergone by all children can be found in Figure 2.

Of the 25 parents who completed the survey, 18 (72%) reported their child as having undergone orthopaedic surgery within the last 2 years.

From the qualitative interviews, four themes were identified from the parents’ descriptions of their experiences through their child’s surgery: (1) preparing and being prepared, (2) feeling known and recognized, (3) knowing and advocating for your child, and (4) feeling stressed and coping. These themes were further expanded to include survey findings on a number of aspects described below.

### 3.1. Theme 1: Preparing and Being Prepared

Parents described preparation for surgery as a process, often starting years in advance. At times, this process began with the identification of their child’s prospective need for orthopaedic surgery or making the decision to proceed. Parents with children at each end of the GMFCS spectrum described how having a warning about surgery allowed them to mentally prepare ahead of time.

“I guess because the whole way along we had an idea that, you know, yes his bone was shifting and eventually it will be in such a position where it will be out so much that this would be how we would fix it.”

(Parent 3)

In describing the decision to pursue surgery, parents expressed the desire to give their child every advantage, an opportunity for independence and comfort, and meet their future needs. When asked to select what motivated those that sought surgery for their child, 11/12 parents surveyed in Group A indicated it was “to improve walking”, while in Group B, 11/13 parents indicated it was “to avoid future problems”. In both groups, the second most common motivation was “to improve quality of life”.

The burden of decision making felt by parents was also identified. Some described the prospect of introducing discomfort and pain to the child’s status quo as a struggle. One parent described the acutely felt risk of making a decision that they or their child may later regret.

“I’m thinking, am I doing [this] decision on my own? Is it right for [my child], or wrong? So, that was always my hardest part … you know, doing this and thinking, oh my God, I hope I’m not making a mistake ….”

(Parent 4)

Parents relied on external guidance and information in deciding and preparing for surgery. Both Groups A and B indicated relying primarily on their child’s healthcare practitioners, with most (23/25) relying “extremely” (17) or “very much” (5) on the surgeon and “extremely” (9) or “very much” (10) on their child’s physiotherapist (PT). While all but one (Group B) parent surveyed indicated they agreed that they were given all information needed to make the decision for their child to have surgery, several of the parents interviewed chose to gather information and reassurance elsewhere. For some, this came in the form of second opinions and, for others, through information from other parents. These secondary sources providing needed reassurance in some cases while introducing potential confusion or the need for caution in others.

“Having the conversations with another family who had been through the surgery was helpful, and so I don’t know if there’s ways to pair people up… At the same time, I mean, you don’t want to scare somebody, because you get, you know, we had a very positive experience, and I would hate to give somebody some ideas [and] then their child not have a similar experience.”

(Parent 1)

Similarly, surveyed parents reported a varied reliance on alternative information sources, including other parents and the Internet, with nearly half indicating that they did not rely at all on these sources.

Several parents interviewed identified feeling prepared and organized to be the most important concern prior to surgery. From basic logistics of identifying, accessing, and setting up needed equipment to details such as what undergarments would fit over a cast, receiving information was described as having considerable impact. Parents relied greatly on rehabilitation professionals (PT and occupational therapist (OT)) and nurses to navigate preparing for their child’s surgery. One parent also identified receiving essential practical advice not found elsewhere from a fellow parent.

“You know it’s somewhat daunting, that you don’t know what you don’t know and so having, you know, the resources there to help… I think that was probably the most helpful, because, yeah, you don’t want to be in a situation where it’s Saturday night, every store’s closed and you’re trying to figure out how to manage, you know, your child that is helpless at this point, in a cast. So, yeah, that was I think the most beneficial.”

(Parent 1)

When asked whether they felt prepared for their child’s surgery, most parents surveyed in Groups A (10/12) and B (9/13) indicated that they completely agreed or agreed, while one parent indicated they received inadequate communication and guidance.

### 3.2. Theme 2: Feeling Known and Recognized

For most parents, the perception of support was, in part, attributed to care provided by practitioners. Some reported finding value in the information, help, and direction provided to parents. Others related having their needs identified or anticipated, describing the relief of feeling taken care of by healthcare practitioners. Several referred to receiving “personalized care”, in which there was a feeling of being known and recognized by the healthcare team. Relationships with their child’s healthcare practitioners were important to parents, with one describing the “comfort of knowing the people, and just feeling that [my child] is in good hands” (Parent 3), and the impact this had on their child’s comfort in the hospital environment.

“I think for the child it’s really important that they have that … feeling that they belong somewhere, and that people recognize them, so yeah, I think it is comforting, because we are here enough, you know?”

(Parent 1)

Parents of children at different GMFCS levels also recognized the ways in which their children were included. For some, this meant children were given a participatory role in decision making either by being directly included in conversations about surgery or by signing their own consent forms. For others, this happened through the communication practices and the actions of practitioners.

“You guys all include [him] and talk to [him], and he is the patient, and it is being done for him, and it’s not just that he’s a chair, you know, like just a fixture in the room.”

(Parent 3)

The absence of familiarity was conversely found to have a negative impact. A parent recounted an incident in which a detailed explanation of an upcoming procedure left the parent needing to comfort and reassure an anxious and upset child. What was felt to be a failure to recognize the personality and emotions of their child led to conflict between the parent and healthcare practitioner.

“They don’t know the child’s personality, right? I mean, for example… if we expect you or we expect [someone else] or whoever is there, once they’ve seen [her] for one visit, two visits, they know that [child’s] personality. And there are ways to ask the questions to her, the way she takes it.”

(Parent 6)

When asked if they felt the healthcare team recognized and knew their child, all but one parent surveyed indicated they completely agreed or agreed. Familiarity also enabled parents to avoid repeating long and complicated medical histories every visit. Parents appreciated being able to “pick up where you left off” and “not have to start at the very beginning” (Parent 1) each visit. Finding a familiar face that knew their child’s surgical history and had expertise in managing their post-operative needs allowed for expedited care.

“He knew [her] well, he’s been part of her surgery, and so he dealt with the casts and what we needed to have done right away, which … was great because I’m not sure the emergency room doctor was necessarily prepared to deal with it, so having just bypassed him made it easier for us, so we didn’t have to wait to see multiple people, you know, spend hours in emergency.”

(Parent 1)

The support and familiarity identified by parents was most often with reference to “the team” rather than any individual or discipline. Parents described a variety of medical specialists, rehabilitation professionals, nurses, and other healthcare practitioners involved in their child’s care throughout the process, as shown for surveyed parents in Table 2. One parent described the benefits of having access to multiple professionals who communicated with one another, all familiar with their child’s history and status. Another described the team of professionals working with their child’s surgeon as “that net underneath [the surgeon] that … just catches everything else.”

(Parent 4)

**Table 2:** Healthcare practitioners reported by parents to be involved in their child’s care throughout surgical experience.

Practitioner	Number of Parents (n = 25)
Surgeon	25 (100%)
Physiotherapist	20 (80%)
Nurse	17 (68%)
Occupational therapist	13 (52%)
Pediatrician	7 (28%)
Family doctor	6 (24%)
Social worker	4 (16%)
Dietician	3 (12%)
Psychologist	1 (4%)
Child life specialist	1 (1%)

Most parents reported that the involvement of their child’s home community rehabilitation team was important to their preparation and recovery. However, when asked whether they agreed that their child’s community PT or OT were included in the preparations for surgery, responses from surveyed parents varied. Most parents in Group A (8/12) indicated they completely agreed or agreed, while others (3/12) somewhat or completely disagreed. In Group B, less than half of parents (5/13) completely agreed or agreed, and less than a quarter (3/13) completely disagreed.

### 3.3. Theme 3: Knowing and Advocating for Your Child

Parents identified parenting a child with CP through surgery as a learning process, guided by their knowledge of their child acquired over years of care. Parents’ experiences with the medical system and familiarity with their child’s daily care and needs provided them with a level of expertise when it came to their child. One parent recounted being the first to identify their child’s dislocated hip.

“We were the ones that … kept saying, you know, when he lays there, his legs are at different lengths and no one seemed to listen to us and… the doctor got out his tape measure and, no they’re the same length, and I’m like, but look, like look! They’re not the same! And it was his physiotherapist, in [the community], [her] who finally was like, no, like when he’s lying, when he’s sitting, when we look, I see what, like, the parents aren’t just making this up.”

(Parent 3)

This expertise also assisted parents in recognizing and helping to manage their child’s pain. For one parent, recognizing how “spoiling” (Parent 2) their child a little bit after surgery and using distraction tools, such as videogames, was found to be superior to pain medication. Another parent described “playing the doctor role” (Parent 3) in an attempt to balance the use of pain medication while trying not to over-sedate their child. For two parents, however, identifying that their child was in pain led to conflict with healthcare practitioners, whom they felt were not providing adequate pain relief and did not acknowledge the parent’s understanding of their child’s needs in the post-operative period.

“I know I’m not a medical professional, but at the same time, like I said, you know your child. You know that … they’re pretty tough cookies, and so clearly, they’re not feeling well, so, you know, just do something to help them!”

(Parent 1)

Both interviewed and surveyed parents indicated feelings of trust towards their healthcare practitioners, with one parent reporting, “I would say it was 100%” (Parent 5). However, all the parents interviewed also identified the need to advocate for their child. Several parents recalled incidents where they felt their child’s needs were unrecognized or ignored by healthcare practitioners, compelling parents to speak up on their behalf. Parents described a learned vigilance, often a reaction to past negative experiences related to their child’s care. One parent described that “sometimes you need to push a little” when not receiving needed information rather than worrying and waiting for follow-up. “A little bit, yeah … because we know what we must do, to get what [he] needs. It’s not about us, it’s about [our child]” (Parent 2). All but one parent surveyed indicated that they completely agreed or agreed when asked whether they could trust their child’s healthcare practitioners. When asked whether they felt their knowledge of their child’s needs were recognized, most parents surveyed in Groups A (10/12) and B (10/13) indicated that they agreed.

### 3.4. Theme 4: Feeling Stressed and Coping

“It’s emotionally exhausting” (Parent 2), recalls one parent about coping through their child’s surgery. Parents described experiencing a range of emotions, both positive and negative, throughout the length of the surgery process. For one, coming to terms with the prospect of surgery left a burden of confusion and guilt.

“You’re like, oh, you know, is it because he’s not horseback riding that he got so weak in his spine and now his back is all gimped? And so, you partly sort of think, well, you stopped it because of his hip surgery and we didn’t get back into it because of money and, you know it’s, you feel like a game.”

(Parent 3)

For some, seeing their child go through the procedure was a painful experience filled with anxiety and fear. Several parents described the difficulty in seeing their child in pain and having no control to be able to help them. Waiting for word while the child was in the operating room was a time of worry and uncertainty. One parent described watching the clock, counting down to the time at which they might expect to get word of their child.

“[The surgeon] comes out and said, yup, your daughter is fine. Everything went good … [The surgeon] leaves. It’s 2 hours, 2 and a half hours, 3 hours. Okay, [the surgeon’s] already out. [The surgeon’s] already out. How come I don’t get a call? It’s 3 hours. As a mom, okay, is my daughter alive?”

(Parent 5)

The times in which parents did not know what was happening with their child were stressful, even when informed that their child was safe. Parents always felt responsible, and being separated from their child was particularly difficult. One parent described a keen awareness of points along the surgery experience in which their child was most vulnerable and feeling helpless when separated at these times.

“The nurses are a little bit more experienced … But at the same time, they don’t know him at all, and so that’s where I get crazy, is waiting to get into [the recovery unit] once I know he’s out of the operating room. So, it’s that uncontrolled environment.”

(Parent 3)

When asked whether their child’s surgery was a stressful experience, more than half of parents in Group A (8/12) and most parents in Group B (12/13) agreed. However, a similar number of parents in Group A (7/12) and in Group B (8/13) indicated that the surgery was not a negative experience.

A positive attitude was a strategy for coping with stress for several parents. For two parents interviewed, this was found to be important in approaching relationships with team members, in helping to create a positive dynamic, and modelling positive behaviour for their child. Positivity was also used in encouraging both the parent and child through a difficult recovery from surgery. For the parent of a child at GMFCS level I, this meant reminding their child of the prospect of not needing to use a brace in the future, and for another at GMFCS level IV, it meant focusing on the possibilities that surgery opened up for their child. Parents acknowledged their role in making their child’s surgery a success and recognized the importance of having the right mindset, being prepared for the unexpected, and learning to adapt.

“The outcome is out of everybody’s hands. You can’t, the surgeons can predict a certain amount, but you can’t, you know, it’s not the Holy Grail answer to everything.”

(Parent 3)

Nearly all parents interviewed found that the surgery experience itself was “not as bad as expected”. This was also true for parents surveyed when asked whether they agreed with this statement, with 7/12 in Group A and 9/13 in Group B at least somewhat agreeing. However, 6/12 parents in Group A and 5/13 in Group B felt their child’s recovery was longer than expected.

In reflecting on the stress and challenge of surgery, parents of children at each GMFCS level identified finding their own meaning in the overall experience. For some, this was found in observing the impact of the surgery results on the child’s life. For the parent of the child at GMFCS level II, seeing their child excited when dropped off at school, where once this was met with tears and tantrums, “really makes you feel good about it” (Parent 6). For others, this was more of a spiritual or existential understanding. For one parent, meaning was found in what their child was felt to bring to the family and the lives of others.

“I sort of feel like, for [our child] is here to help me learn and our family learn about, you know, living a happier, better life. But he’s also helping all the team, the people around him and so … if his presence is to, you know, help other people feel more comfortable around kids like that, or help the doctors try new techniques … I feel like he’s helping everybody in their development and their learning too.”

(Parent 3)

## 4. Discussion

The aim of this study was to explore the experience of parents of children with CP across the GMFCS levels undergoing orthopaedic surgery. At the study centre, in addition to orthopaedic surgeons, an interprofessional team approach to care has been developed and implemented, in which care is managed and supported by rehabilitation and nursing professionals, with robust knowledge of evidence-based care of children with CP. This approach prioritizes the collaborative assessment and management of patient needs, with an emphasis on providing education for families, preparation for surgery, and coordination of services. This is in keeping with the principles of family-centred care [23], in that collaboration is not within team members alone but is routinely extended to families to, in effect, provide flexible service delivery, individualized according to the needs of the child, parents, family, and circumstance [21,38].

Study participants highlighted the importance of feeling prepared for surgery. In an earlier qualitative study of twelve parents of nine children with CP undergoing orthopaedic surgery, parents were found to feel helpless and unprepared in the absence of adequate information about their child’s situation and care [39]. More recently, the importance of preparation was highlighted by 14 parents and family members of children with CP undergoing scoliosis surgery, in which it was identified as a key to coping [40]. In the current study, most parents interviewed felt prepared for their child’s surgery. They described a preparation process assisted by healthcare providers, beginning with the prospect of surgery for their child, followed by receiving and seeking information to make the decision for surgery and further direction through the details and logistics of what was to come. The healthcare team, not a particular individual, was identified as providing support, suggesting that the interdisciplinary care model utilized in the study centre positively influenced the parents’ experience. Parents also noted seeking advice outside the healthcare team, most notably from other parents. This finding suggests a possible need for the exploration of the role of peer support for parents, prior to a child’s orthopaedic intervention [41].

The impact of relationships with healthcare practitioners on caregivers has previously been described. Iversen et al. [42] identified that when parents observed interactions between practitioners and their children in which interest was shown and time taken to connect with a child, trust was also established with the parent. Hayles, Harvey, et al. [43] reported parents’ preference for partnership with practitioners when addressing the healthcare needs of their child. Similarly, for parents in the current study, feeling that both their children and they were known and recognized by practitioners over frequent medical visits and treatments fostered feelings of trust and confidence in those caring for their children. Feeling recognized for their knowledge and role in meeting the healthcare needs of their child has also been identified as important for parents in fostering partnership with practitioners [43]. Parents of non-verbal children, for example, have been recognized to be familiar with and sensitive to their child’s bodily signs that indicate pain or other sensations [42]. This has led parents again to experience ambivalence towards their child’s healthcare practitioners, both trusting and doubting their ability to recognize and meet their child’s needs [42,44] and consequently leaving parents feeling the need to advocate on their child’s behalf. The desire of parents to be recognized by health professionals for their expertise in their child’s needs through surgery was also noted by Stewart et al. [40]. Our findings support those of Hayles, Harvey, et al. [43], who found that parents felt they knew their children best and that they were ultimately responsible for their children and wished to work with practitioners in determining care.

The stress experienced by parents of children with CP has been well described [19,45,46,47]. Parents of children with CP have been found to experience very high stress five times more often than the general population [45]. A child’s medical treatment or surgery has been described as a time of emotional strain and vulnerability [39,48]. Researchers have suggested that parental stress is associated with the child’s level of motor impairment, with parents of children with greater impairments experiencing greater stress [19,49], though this relationship has not consistently been found [45,50,51,52]. In this study, participants surveyed in both groups, across all GMFCS levels, indicated surgery to be a stressful experience. This suggests the stress experienced by parents may not have been determined by a child’s functional mobility or the type of procedure performed. The surgical experience, including the difficulty with seeing your child in pain [45] and uncertainly over safety, may be felt similarly by all parents, regardless of GMFCS or surgical procedure. Parents also indicated a high level of coping, using strategies such as maintaining a positive outlook. Such elements of resilience, in difficult times, have previously been identified in parents of children with CP [48]. Research suggests that the determinants of parent stress may be multifactorial [45,47,53] and dependent on a family’s intrinsic level of resilience [19]. This is particularly relevant for providers of surgical care to children with CP and their families, where an individualized approach to care including psychosocial support for both child and family may be most appropriate [19,54].

Interestingly, most parents identified their child’s surgery as being a stressful experience, but less than half identified it as having been a negative one. Our findings suggest that the relationships established with the interdisciplinary orthopaedic healthcare team and the feeling of preparation supported parents through the surgical experience. As many children with CP require ongoing orthopaedic interventions over the course of their development, a more consistent approach to care, as is provided at the study centre, is needed rather than the typical episodic delivery of surgical care [43,55]. This includes regular follow-up visits for the child and family with the surgeon and/or therapy or nursing team, even in the absence of immediate surgical plans. In addition to monitoring for orthopaedic complications and the proactive management of the surgical needs of children, regular visits offer an opportunity for education and building relationships between healthcare team members, the child, and parents or caregivers. The value of a “therapeutic” or “working alliance” [56] between the health provider and parent has long been recognized for its influence on positive outcomes in the care of children with disabilities [57] and on the satisfaction of parents in the experience of care [58]. Such a partnership, however, is only one element of a family-centred care model, which may not satisfy parents or serve all of the broad and unique needs of children and families [57,59]. While beyond the scope of this study, future exploration of this interdisciplinary model of care, its comparison to other models, and how it may work in concert with family-centred care is warranted [60].

While it is conceivable that the interprofessional team may have contributed to most parents finding the experience of their child’s surgery to be better than originally anticipated, many parents did not find this to be true of their child’s subsequent recovery period. This suggests a need for greater education of parents regarding what to expect following surgery. In a cross-cultural exploration of the perspectives of Canadian and Indian parents of children with CP, the desire for more information from providers in general, including relating to their child’s prognosis and avenues for intervention, was identified across study participants [61]. A need for increased availability of services for children was also recognized by parents [61]. However, cultural differences were found in the degree to which parents advocated for information and services on behalf of their children [61]. Given that impairments in a child’s communication and behaviour and their experiences of pain have been demonstrated to be associated with higher parental stress [45] and that these factors can further challenge the inherent difficulties of a child’s post-operative course, it may also underscore a need for greater support for parents. This includes not only help throughout the aftermath of their child’s surgery and hospitalization but also ensuring accessible rehabilitation to help guide and facilitate both children and families through the recovery process. The delivery of such support, however, also requires an effective family-centred approach to rehabilitation [61], one that recognizes the socioeconomic, education, and cultural experiences of individual families [38,61,62].

### Study Limitations

There are limitations to this study. Saturation of data may not have been achieved due to the small number of only 6 participants in the interview strand, and the small convenience sample of 25 survey participants limits the transferability of findings, particularly in relation to comparisons of parent experiences with children across functional levels. Additionally, the small sample size may not be representative across socioeconomic and cultural contexts. Sampling also occurred at a single tertiary care centre located in an urban setting. While the institution provides services for children and families across a broad geography, it is possible the sample did not represent the diversity of challenges experienced by those in rural vs. urban environments. The exclusion of parents with limited English proficiency also restricts insight into the experiences of a potentially vulnerable population and also limits generalizability of findings. The length of time from their child’s surgery could have resulted in inaccurate recollection of the experience for parents. However, all parents in the qualitative strand and 72% of survey respondents participated within 2 years of their child’s surgery. For some parents, it is possible the proximity of the researcher performing the interviews to the clinical setting could have resulted in withholding or under-reporting by some parents in sharing their experiences. We also acknowledge that there was an extended period between the data collection and reporting of these results. However, we feel that this study highlights the experience of parents as a consideration in surgical planning and presents important directions for future exploration that are clinically relevant today. These future research opportunities include the comparison of the parent experience across different care models and the role of secondary supports such as parent peer groups.

## 5. Conclusions

This study explored the experiences of parents of children with CP across the GMFCS levels requiring orthopaedic surgery and identified four themes: (1) preparing and being prepared, (2) feeling known and recognized, (3) knowing and advocating for your child, and (4) feeling stressed and coping. This is the first study, to our knowledge, that also considered these experiences across the GMFCS spectrum, with parents reporting similar experiences regardless of their child’s functional mobility. These findings highlight some of the needs of parents and suggests opportunities for collaboration with families in the development and design of service delivery approaches within this population [60]. Approaches to care that foster partnerships and individualized support may more directly meet the needs of parents and families during a time of heightened stress [63,64]. Interprofessional care offers an opening for improved experiences for families over the course of a child’s medical care and development [55].

## Figures and Tables

**Table 1:** Characteristics of children and parents in the interview group, survey Group A, and survey Group B.

Characteristic	Interview Group	Survey Group A	Survey Group B
Number of parents (mothers/fathers)	6 (5/1)	12 (11/1)	13 (12/1)
GMFCS level (# of children)	I (1), II (1), III (1), IV (1), V (1)	I (1), II (7), III (4)	IV (8), V (5)
Number of Children in Family			
1	1	4	8
≥2	5	8	5
Parent Relationship Status			
Single	1	2	0
Married/domestic Partnership	5	9	10
Separated/divorced	0	1	3
Parent Work Status			
Full-/part-time employment	3	6	5
Homemaker	3	6	8
Parent Age			
	Mean: 44.7	Range: 25–64	Range: 25–54
Child Age			
	Mean: 10.4	Range: 4–18	Range: 4–18

## Data Availability

In consideration of participant privacy, the data presented in this study are available from the corresponding author, M.J. (mjuricic@cw.bc.ca), upon reasonable request.

## Supplementary Materials

The following supporting information can be downloaded at: https://www.mdpi.com/article/10.3390/children12101411/s1, File S1: Parent Interview Script.
