# Correction: Juricic et al. Children with Cerebral Palsy Across the Gross Motor Function Classification System Levels Requiring Orthopaedic Surgery: The Lived Experiences of Parents. *Children* 2025, *12*, 1411

**DOI:** 10.3390/children12121630

**Published:** 2025-12-01

**Authors:** Maria Juricic, Stacey D. Miller, Emily K. Schaeffer, Kishore Mulpuri, Lesley Bainbridge

**Affiliations:** 1 Department of Orthopaedic Surgery, BC Children’s Hospital, Vancouver, BC V6H 3V4, Canada; 2 Department of Physical Therapy, University of British Columbia, Vancouver, BC V6T 1Z3, Canada; 3 BC Children’s Hospital Research Institute, Vancouver, BC V5Z 4H4, Canada; 4 Department of Orthopaedics, University of British Columbia, Vancouver, BC V5Z 1M9, Canada

**Error in Table**

In the original publication [1], there were some mistakes in Table 1 as published. Table 1 contained several inaccuracies in the demographic data.

The corrected Table 1 appears below. The authors state that the scientific conclusions are unaffected. This correction was approved by the Academic Editor. The original publication has also been updated.

## Figures and Tables

**Table 1:** Characteristics of children and parents in the interview group, survey Group A, and survey Group B.

Characteristic	Interview Group	Survey Group A	Survey Group B
Number of parents (mothers/fathers)	6 (5/1)	12 (11/1)	13 (12/1)
GMFCS level (# of children)	I (1), II (1), III (1), IV (1), V (1)	I (2), II (7), III (3)	IV (5), V (8)
Number of Children in Family			
1	1	4	8
≥2	4	8	5
Parent Relationship Status			
Single	1	2	0
Married/domestic Partnership	5	9	10
Separated/divorced	0	1	3
Parent Work Status			
Full-/part-time employment	3	6	6
Homemaker	3	6	7
Parent Age			
	Mean: 44.7	Range: 25–64	Range: 25–54
Child Age			
	Mean: 10.4	Range: 4–18	Range: 4–18
