# Supplementary material for: Children with Cerebral Palsy Across the Gross Motor Function Classification System Levels Requiring Orthopaedic Surgery: The Lived Experiences of Parents
Source: Children (Basel). 2025 Oct 18;12(10):1411. doi: 10.3390/children12101411 (PMC12562333; doi:10.3390/children12101411)
Supplement: Supplementary file 1 [file children-12-01411-s001.zip › children-3883498-supplementary.pdf]

Children with CP Across GMFCS Levels Requiring Orthopaedic Surgery:
The Lived Experiences of Parents

Parent Interview Script

Introduction & Consent

☐ Introduce self.
☐ Explain study including aims.
☐ Read through information sheet and consent form together.
☐ Answer questions and sign consent (parent to sign with research associate).
☐ "Please feel free to stop me at any time as we go, if you need any clarification, or if you feel uncomfortable answering any questions. If you become stressed or upset, you may also stop the interview and should you wish I can refer you to someone to talk to."

Demographic Information

☐ "I would like to ask you a few questions to learn a bit more about you and your family."
☐ Explain that the purpose of the following questions is to help me to understand what other factors might contribute to the parent experience and to assist in the data analysis.

<u>Child</u>:
- Gender M / F
- Age of child:
- Diagnosis:
- Related medical conditions:
- Gross Motor Function Classification System level (Use Family Report Questionnaire if not known):

<u>Parents</u>:
- Mother/Father/other (circle one):
- Marital status:
- Age:
- Occupation:
    - Mother:
    - Father:
- Highest level of education:
    - Mother:
    - Father:

<u>Family</u>:

- Who lives at home?
- Siblings & ages:
- Funding sources: At Home Program? Extended benefits? Other?

Parent & Child

- ☐ I would like to ask you about what is involved in caring for your child on a typical day. Can you describe that for me?
- ☐ What support do you have in caring for your child?
    - o How well do you feel supported?
    - o If not well, what additional supports do feel you need?
- ☐ From whom do you usually get health/medical information to help meet your child's needs?
- ☐ What other sources of information do you access? (Prompts – website, library, etc.)

Surgery

- ☐ What have been the surgical needs of your child?
- ☐ I would like to ask you about the time leading up to surgery.
    - o What motivated you to make the decision for your child to have surgery?
    - o How did you come to the decision – what steps were involved?
    - o How did you access all the information you needed?
    - o How were the goals of surgery decided?
        - o How were you involved in this?
- ☐ How did you prepare for surgery?
- ☐ What, if any, kind of extra support did you put in place?

Surgery process

- ☐ As a parent, how did you cope through your child's surgery process?
- ☐ Which specific health care professionals (HCPs) were involved in care of your child throughout the surgery process?
- ☐ What were the positive aspects of working with HCPs through the surgery process?
- ☐ What were some of the aspects that were not so positive?
- ☐ Overall, how would you describe your relationship with your health care professionals?
- ☐ Overall, how would you describe your child's relationship with his/her health care professionals?

Reflection on experience

- ☐ What effect has surgery had on your child?
- ☐ What effect has surgery had on your family?
- ☐ Looking back, how did the actual experience compare to what you anticipated?
- ☐ What helped?

☐ What made it harder?
☐ Is there anything that you would have liked to know then, that you know now?
☐ What would you tell another family preparing for surgery?

Summary
☐ Is there anything I didn't ask you that you wished I had?

GMFCS Family Report Questionnaires:
https://canchild.ca/wp-content/uploads/2025/03/GMFCS_Family.pdf?license=yes
